# Bee Venom Inhibits *Porphyromonas gingivalis* Lipopolysaccharides-Induced Pro-Inflammatory Cytokines through Suppression of NF-κB and AP-1 Signaling Pathways

**DOI:** 10.3390/molecules21111508

**Published:** 2016-11-10

**Authors:** Woon-Hae Kim, Hyun-Jin An, Jung-Yeon Kim, Mi-Gyeong Gwon, Hyemin Gu, Jae-Bok Park, Woo Jung Sung, Yong-Chul Kwon, Kyung-Duck Park, Sang Mi Han, Kwan-Kyu Park

**Affiliations:** 1 Department of Pathology, College of Medicine, Catholic University of Daegu, 33, Duryugongwon-ro 17-gil, Nam-gu, Daegu 42472, Korea; kimwoonhae@cu.ac.kr (W.-H.K.); ahj119@cu.ac.kr (H.-J.A.); jy1118@cu.ac.kr (J.-Y.K.); daldy99@naver.com (M.-G.G.); asdf8760@naver.com (H.G.); jbpark@cu.ac.kr (J.-B.P.); wjsung@cu.ac.kr (W.J.S.); kyc1855@hanmail.net (Y.-C.K.); 2 Department of Dermatology, College of Medicine, Catholic University of Daegu, 33, Duryugongwon-ro 17-gil, Nam-gu, Daegu 42472, Korea; gdpk1217@naver.com; 3 Department of Agricultural Biology, National Academy of Agricultural Science, Rural Development Administration, 300, Nongsaengmyeong-ro, Wansan-gu, Jeonju-si 54875, Korea; sangmih@korea.kr

**Keywords:** periodontitis, *Porphyromonas gingivalis*, lipopolysaccharides, bee venom

## Abstract

Periodontitis is a chronic inflammatory disease that leads to destruction of tooth supporting tissues. *Porphyromonas gingivalis* (*P. gingivalis*), especially its lipopolysaccharides (LPS), is one of major pathogens that cause periodontitis. Bee venom (BV) has been widely used as a traditional medicine for various diseases. Previous studies have demonstrated the anti-inflammatory, anti-bacterial effects of BV. However, a direct role and cellular mechanism of BV on periodontitis-like human keratinocytes have not been explored. Therefore, we investigated the anti-inflammatory mechanism of BV against *P. gingivalis* LPS (PgLPS)-induced HaCaT human keratinocyte cell line. The anti-inflammatory effect of BV was demonstrated by various molecular biological methods. The results showed that PgLPS increased the expression of Toll-like receptor (TLR)-4 and pro-inflammatory cytokines, such as tumor necrosis factor (TNF)-α, interleukin (IL)-1β, IL-6, IL-8, and interferon (IFN)-γ. In addition, PgLPS induced activation of the signaling pathways of inflammatory cytokines-related transcription factors, nuclear factor kappa-light-chain-enhancer of activated B cells (NF-κB) and activator protein 1 (AP-1). BV effectively inhibited those pro-inflammatory cytokines through suppression of NF-κB and AP-1 signaling pathways. These results suggest that administration of BV attenuates PgLPS-induced inflammatory responses. Furthermore, BV may be a useful treatment to anti-inflammatory therapy for periodontitis.

## 1. Introduction

Periodontitis, one of the most common inflammatory diseases in humans, is a chronic inflammatory disease that leads to destruction of tooth supporting tissues [1]. Although not fatal, it can cause tooth loss and marked reduction in quality of life. It is well documented that periodontitis is initiated by microbes embedded in subgingival dental plaque (biofilm) and related to complex interactions of bacteria with the host [2,3]. The development of this disease is a multifactorial process involving interactions between the host and the microorganisms that colonize the oral cavity [4,5].

A gram-negative anaerobic bacterium, *Porphyromonas gingivalis* (*P. gingivalis*), is a major etiological pathogen of severe periodontitis and causes alveolar bone resorption [6]. *P. gingivalis* can locally invade periodontal tissues and evade the host defense mechanisms. In doing so, it utilizes a panel of virulence factors that cause deregulation of the innate immune and inflammatory responses [7]. *P. gingivalis* adheres to, invades, and replicates within human epithelial cells and can modulate cell signal transduction pathways, directing its uptake by gingival epithelial cells [8].

Gingival epithelial cells function as an innate physical barrier to prevent invasion by periodontal bacteria [9]. Nevertheless, several strains of periodontal bacteria—including *P. gingivalis*, *Tannerella forsythia*, and *Treponema denticola*—have been detected among epithelial cells obtained from periodontal pockets, gingival crevices, and buccal mucosa specimens collected from both periodontitis patients and subjects with healthy gingivae [10,11]. The perturbation of epithelial cells by bacteria is the first stage in the initiation of the inflammatory and immune processes, which eventually cause destruction of the tissues surrounding and supporting the teeth, ultimately resulting in tooth loss [12].

Oral keratinocytes reside at the gingival sulcus and respond to bacterial challenge via Toll-like receptor (TLR)s. Their activation in keratinocyte is the first line of defense in immune system and produce a variety of pro-inflammatory cytokines and chemokines [13]. These cytokines—including interferon (IFN)-γ, interleukin (IL)-1β, IL-8, and tumor necrosis factor (TNF)-α—are produced by human keratinocytes and activate neutrophils and macrophages [14,15].

Clinical intervention in periodontitis is primarily mechanical and involves scaling of the subgingival tooth surface to remove the plaque biofilm. In the event of significant loss of tooth-supporting tissues, surgical procedures are sometimes carried out to restore the periodontal architecture and stimulate regeneration of supporting tissues [16]. In this context, it should be noted that conventional periodontal treatment is often not sufficient by itself to control destructive inflammation and many patients develop recurrent disease [17,18]. Several anti-inflammatory interventions have been tested in preclinical animal models of periodontitis, including non-human primates, which share key clinical, microbiological, and immunohistological features with the human disease [19,20,21,22]. However, there is not yet an obvious anti-inflammatory agent for periodontitis. Although antibiotics are conventional therapeutic agents for bacterial infection, they have been known to induce some side effects. Recently, reports have suggested that the natural toxin bee venom (BV) has anti-bacterial and anti-inflammatory effects [23,24].

BV therapy has been used in oriental medicine for the relief of pain and the treatment of inflammatory diseases, such as rheumatoid arthritis and multiple sclerosis [25,26]. Previous studies have demonstrated the anti-inflammatory effect of bee venom in rheumatoid arthritis, allergic asthma, and atherosclerosis [25,27]. However, there has not yet been a robust trial to prove a therapeutic effect of bee venom in periodontitis. In the present study, the anti-inflammatory properties of bee venom were investigated in a periodontitis model stimulated by *P. gingivalis* lipopolysaccharide (PgLPS), using a human keratinocyte cell line.

It has been established that the host inflammatory response against bacteria and their virulence factors is the basis for understanding the pathogenesis of chronic periodontitis [28,29,30,31,32,33]. The destructive response of the organism is associated with an increased expression of inflammatory cytokines in the tissue [28,29,32,34,35]. Further, it has been shown that the gingival tissue in patients with chronic periodontitis contains larger amounts of pro-inflammatory cytokines—such as IL-1, 6, 8, TNF-α, and other chemical mediators of inflammation—in comparison with those of healthy individuals [32].

Among these cytokines, IL-6 and IL-8 are considered to be crucial pro-inflammatory factors correlated with the initiation of periodontitis [36]. IL-6 is responsible for modulating the inflammatory cascade associated with chronic periodontitis [37]. IL-8 is a major chemokine involved in neutrophil infiltration into periodontal lesions leading to exacerbation of the inflammation in periodontal tissue [38]. TNF-α is a multifunctional cytokine involved in the regulation of immunity and inflammation [39]. Recent studies have indicated that TNF-α, IL-1β, and IL-8 accelerate skin inflammation in mice. Also, these cytokines and chemokines were shown to modulate inflammatory responses in keratinocytes and monocytes [40,41].

A multitude of studies have shown that *P. gingivalis* modulates the innate host defense functions by subversion of IL-8 secretion, complement activity, and Toll-like receptor (TLR)-4 activation [16]. Furthermore, bacteria stimulate the production of pro-inflammatory cytokines via TLR-2 and TLR-4 [42,43]. Therefore, some pro-inflammatory cytokines and TLR-4 were researched to determine the expression level during PgLPS stimulation in the present study. Several studies have demonstrated that keratinocytes and monocytes induce pro-inflammatory cytokines through a TLR-2-dependent pathway [44,45]. TLRs are expressed by various cells of the innate immune system, such as monocytes, macrophages, and granulocytes [46]. Activation of TLRs promotes the production of pro-inflammatory cytokines, prostaglandins, leukotrienes, and chemokines [47]. Ten human TLRs, with different ligand specificities, have been identified. TLR-4 is associated with cluster differentiation (CD)14 and is mainly involved in mediating lipopolysaccharide (LPS)-induced cellular signaling of gram-negative bacteria [48]. By contrast, TLR-2 recognizes lipopeptides from gram-positive bacteria and contributes to the innate immune response of human epidermal keratinocytes [49].

During inflammatory reactions, TLR activation results in the activation of the mitogen-activated protein kinases (MAPK), protein kinase B (Akt), the nuclear factor kappa-light-chain-enhancer of activated B cells (NF-κB), and activator protein (AP)-1 signaling pathways. These pathways modulate inflammatory gene expression, which is crucial in shaping the innate immune response within the inflammatory disease [50,51,52].

The aim of this study was to evaluate the inhibitory effect of BV on the expression of inflammatory cytokines and TLR-4 in PgLPS-stimulated keratinocytes. In the present study, the expressions of inflammatory cytokines, TLR-4, NF-κB, extracellular signal-regulated kinase (ERK), Akt, and c-Jun (which is major component of AP-1) were increased in the PgLPS-treated keratinocytes. Their expressions were decreased after BV treatment. The activation of transcription factor AP-1 and NF-κB, known to be related to the expressions of inflammatory cytokines, was also evaluated. Therefore, these results might reveal the potential therapeutic effects of BV as an alternative agent for *P. gingivalis*-induced periodontitis. In addition, the molecular pathogenesis of periodontitis and the anti-inflammatory effects of BV were investigated in the present study.

## 2. Results

### 2.1. Effects of BV on Cell Viability

**Figure 1:**
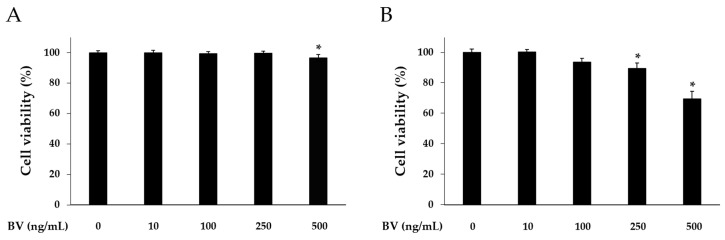
Cytotoxic effects of BV on HaCaT cells. Cytotoxicity of bee venom was determined by CCK-8 assay. HaCaT cells were treated with 1, 10, 100, 250, and 500 ng/mL of BV for 8 h (**A**); and 24 h (**B**). Results are expressed as the means ± SEM of three independent determinations. * *p* < 0.05 compared to the untreated group.

Cell counting kit (CCK)-8 assay was carried out to determine cytotoxicity of BV at different doses. HaCaT cells were treated with 10, 100, 250, and 500 ng/mL of BV for 8 or 24 h. In the 8 h BV treatment, the HaCaT cell viability was reduced at the 500 ng/mL concentration of BV. However, 10, 100, and 250 ng/mL of BV did not alter the HaCaT cell viability (Figure 1). In the 24 h BV treatment, the HaCaT cell viability was reduced at the 100, 250, and 500 ng/mL concentrations of BV. In accordance with this result, BV treatment was performed for 8 h, and 500 ng/mL concentration of BV was excluded in subsequent experiments.

### 2.2. BV Inhibits PgLPS-Induced the Pro-Inflammatory Cytomokines and TLR-4

**Figure 2:**
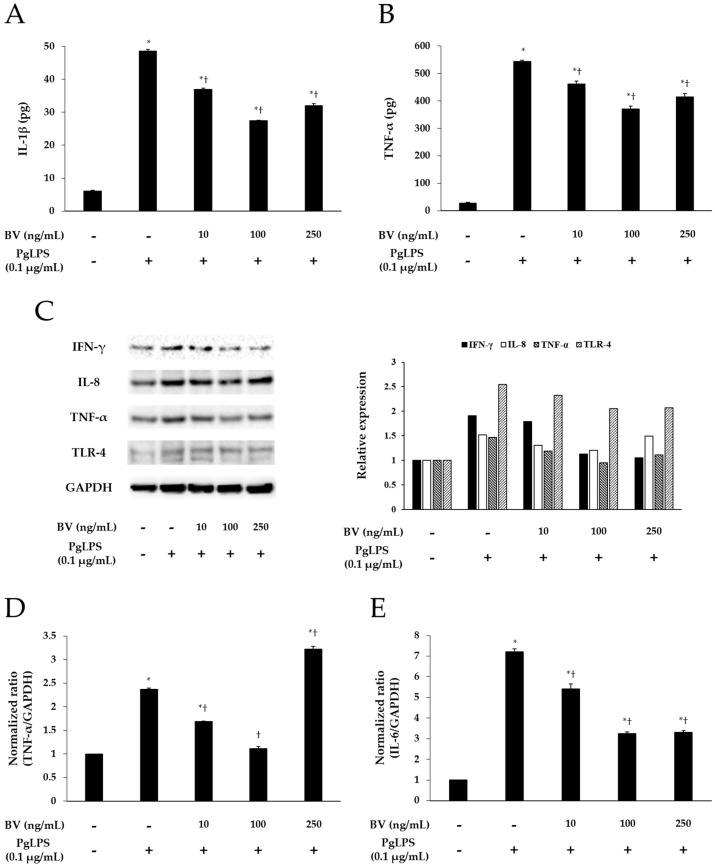

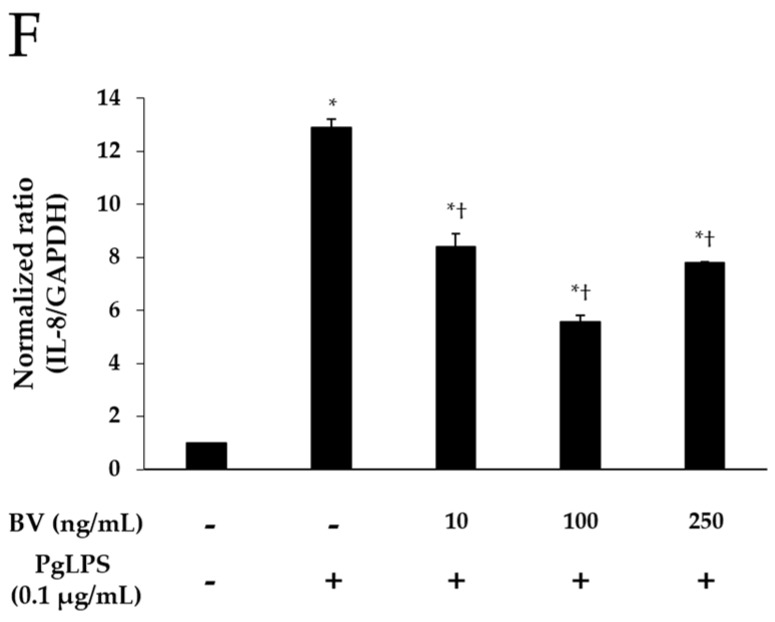
Effect of BV on the expression of pro-inflammatory mediators and TLR-4 in PgLPS-treated HaCaT cells. ELISA results demonstrate that BV suppressed the secretion of IL-1β (**A**); and IL-6 (**B**); (**C**) Western blot analysis showed that BV inhibited the expression of IFN-γ, IL-8, TNF-α, and TLR-4. GAPDH was used to confirm equal sample loading. Results are representative of three independent experiments; (**D**) 10 and 100 ng/mL of BV suppressed TNF-α mRNA expression, and 250 ng/mL of BV encouraged TNF-α mRNA expression; (**E**) IL-6 mRNA expression was inhibited by 10, 100, and 250 ng/mL BV; (**F**) 10, 100, and 250 ng/mL of BV reduced IL-8 mRNA expression. Results are expressed as the means ± SEM of three independent determinations. * *p* < 0.05 compared to the untreated group; † *p* < 0.05 compared to the PgLPS group; +: treated; −: untreated.

To demonstrate the effect of BV on the inflammation, induced by PgLPS, BV was administrated to the PgLPS-treated HaCaT cells. HaCaT cells were pre-incubated with or without BV, and treated with or without PgLPS. After treatment, the culture supernatants were collected for enzyme-linked immunosorbent assay (ELISA). The cells were collected for real-time polymerase chain reaction (PCR) and Western blot. ELISA was employed to examine the effects of BV on the secretion of IL-1β and TNF-α from PgLPS-treated HaCaT cells. PgLPS increased the secretion of IL-1β and TNF-α, compared with normal control (NC), and 10 and 100 ng/mL of BV treatments significantly suppressed the secretion of IL-1β and TNF-α, compared with PgLPS group. However, 250 ng/mL of BV treatment suppressed the secretion of IL-1β and TNF-α less effectively than 100 ng/mL of BV treatment (Figure 2A,B). Thus, these observations suggest that 10, 100, and 250 ng/mL of BV effectively inhibits the secretion of IL-1β and TNF-α in PgLPS-treated HaCaT cells, but the most efficient concentration of BV was 100 ng/mL.

Real-time PCR was used to elucidate the effects of BV on the gene expression of TNF-α, IL-6, and IL-8 in PgLPS-treated HaCaT cells. As shown in Figure 2D–F, PgLPS markedly increased the mRNA expression of TNF-α, IL-6, and IL-8, compared with NC, and 10 and 100 ng/mL of BV treatment significantly suppressed the mRNA expression of IL-6, IL-8, and TNF-α. However, the results of 250 ng/mL of BV treatment were different in each case (i.e., TNF-α, IL-6, IL-8). In the case of TNF-α, 250 ng/mL of BV treatment did not decrease TNF-α mRNA but rather increased TNF-α mRNA, compared with the PgLPS group, whereas 250 ng/mL of BV treatment inhibited IL-6 mRNA similarly to 100 ng/mL of BV treatment. Furthermore, 250 ng/mL of BV treatment inhibited the IL-6 mRNA less effectively than 100 ng/mL of BV treatment. These results show that 10 and 100 ng/mL of bee venom exhibited the suppression of TNF-α, IL-6 and IL-8 mRNA expressions, and 250 ng/mL of BV resulted in the suppression of IL-6 and IL-8 mRNA expressions and the encouragement of TNF-α mRNA expression in PgLPS-treated HaCaT cells.

Western blot results showed the effects of BV on PgLPS-induced pro-inflammatory cytokines, and TLR-4 expressions in HaCaT cells. HaCaT cells expressed IFN-γ, IL-8, TNF-α, and TLR-4 after exposure to PgLPS. On the other hand, BV treatment suppressed the expression of IFN-γ, IL-8, TNF-α, and TLR-4 in different patterns. First, BV suppressed the IFN-γ expression in a dose-dependent manner. In the cases of IL-8, TNF-α, and TLR-4, their expression levels reduced in the 10 and 100 ng/mL of BV, but not in the 250 ng/mL of BV (Figure 2C). These results show that 100 ng/mL of BV is the optimal concentration for the suppression of IL-8, TNF-α, and TLR-4 expressions, and BV significantly inhibits the expression of IFN-γ in PgLPS-treated HaCaT cells.

### 2.3. BV Suppresses Signaling Pathways of Inflammatory Transcription Factors in PgLPS-Treated HaCaT Cells

**Figure 3:**
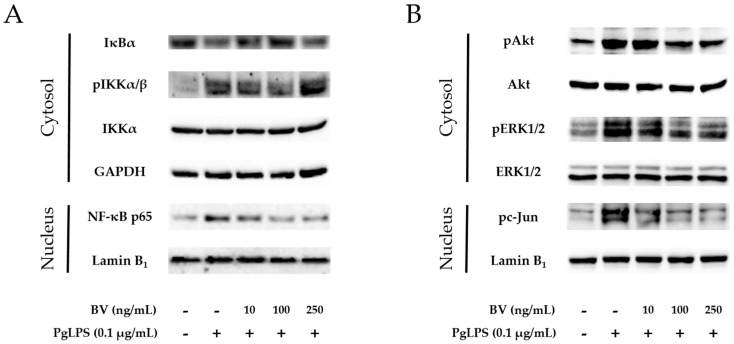
BV inhibits the NF-κB signaling pathway and activation of Akt, ERK1/2 and c-Jun in PgLPS-treated HaCaT cells. (**A**) BV suppressed expression of cytosolic pIKKα/β and nuclear NF-κB, while BV improved cytosolic IκB, compared with the PgLPS-treated group; (**B**) BV suppressed activation of cytosolic Akt, ERK1/2, and nuclear c-Jun, compared with the PgLPS-treated group. GAPDH and Lamin B1 were used to confirm equal sample loading. Results are representative of three independent Western blot analyses. +: treated, −: untreated.

The NF-κB signaling pathway is a central player in a wide variety of chronic inflammatory diseases, and NF-κB inhibitor (IκB) and IκB kinase (IKK) are important proteins in the NF-κB signaling pathway [53]. In this study, to determine the involvement of IKK, IκB, and NF-κB in the anti-inflammatory property of BV, activation of these proteins was examined by Western blots (Figure 3A). Following the administration of PgLPS, increased expressions of cytosolic phosphorylated (p)IKK, nuclear NF-κB were found in the PgLPS-treated cells, while decreased expression of cytosolic IκB. Addition of 10 ng/mL and 100 ng/mL BV reduced cytosolic pIKK, nuclear NF-κB more effectively than 250 ng/mL of BV treatment, and the pattern of IκB expression appeared contrastively.

Activation of Akt and ERK is important in the LPS-induced innate immune response. ERK could play a crucial role to regulate AP-1 transcription factor complex, composed of proteins belonging to the c-Fos and c-Jun. [54]. To determine the effect of BV on the Akt/ERK activation and expression of c-Jun in PgLPS-stimulated HaCaT cells, Western blot was performed (Figure 3B). HaCaT cells expressed pAkt, pERK, and pc-Jun after exposure to PgLPS, compared with the NC group. In contrast, the PgLPS-induced expression of pAkt, pERK, and pc-Jun were inhibited by BV in a dose-dependent manner.

### 2.4. BV Reduces the DNA-Binding Activity of Inflammatory Transcription Factors in PgLPS-Treated HaCaT Cells

**Figure 4:**
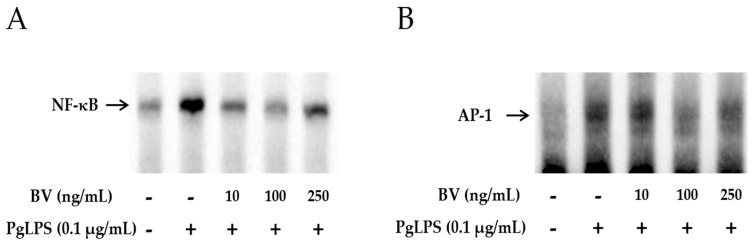

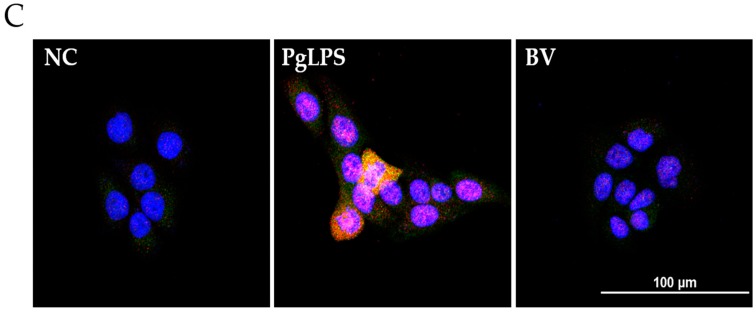
BV reduces the DNA binding activity of NF-κB and AP-1 in PgLPS treated HaCaT cells. EMSA results showed that BV treatment suppresses the binding activity of NF-κB (**A**); and AP-1 (**B**) in PgLPS-induced HaCaT cells. Results are representative of three independent experiments. +: treated; −: untreated; (**C**) BV inhibits the expression of TNF-α and NF-κB in PgLPS-treated HaCaT cells. Representative immunofluorescence images show that 100 ng/mL of BV suppressed TNF-α (labeled with Alexa Fluor 488, **green**) and NF-κB (labeled with Alexa Fluor 555, **red**). The nuclei were labeled with Hoechst 33342 (**blue**). NC: normal control; PgLPS: 0.1 μg/mL of *P. gingivalis* LPS; BV: 0.1 μg/mL of *P. gingivalis* LPS + 100 ng/mL of BV.

To examine whether BV effectively blocks the DNA-binding activity of NF-κB and AP-1, gel electrophoresis mobility shift assays (EMSA) were performed. As shown in Figure 4A,B, the binding activity of NF-κB and AP-1 was increased in PgLPS-treated HaCaT cells. In contrast, this enhancement of binding activity was markedly withdrawn after treatment with 10 ng/mL and 100 ng/mL of BV, while 250 ng/mL of BV treatment suppressed the binding activity of NF-κB and AP-1 less effectively than 100 ng/mL of BV.

In the case of TNF-α and NF-κB, the protein expression levels were examined by Western blot, and the results showed that the optimal concentration of BV was 100 ng/mL in PgLPS-treated HaCaT cells. Furthermore, to determine whether 100 ng/mL of BV affects the distribution of TNF-α and NF-κB in PgLPS-treated HaCaT cells, immunofluorescence labeling was performed (Figure 4C). The distribution of TNF-α and NF-κB in NC displayed little TNF-α and NF-κB expression in the cytoplasm. In contrast, the PgLPS-treated group showed strong labeling intensity of TNF-α in the cytoplasm and NF-κB in the nucleus. In the case of the BV-treated group, the expression of TNF-α and NF-κB was significantly reduced, compared with the PgLPS-treated group. These data suggest that PgLPS induced activation of TNF-α and NF-κB in HaCaT cells, and 100 ng/mL of BV reduced the expression of TNF-α and NF-κB in PgLPS-treated HaCaT cells.

## 3. Discussion

BV and its components induce a variety of immune responses, including both acute inflammatory responses, such as mast cell degradation, and adaptive immune responses. However, the molecular mechanisms by which the innate immune system detects envenomation and initiates inflammatory and allergic responses remain largely unknown [55]. Interestingly, relatively high concentrations (≥2.5 μg/mL) of BV trigger the increase of IL-1β and IL-18 release in human keratinocytes [56]. In contrast, relatively low concentrations (≤100 ng/mL) of BV decreased the inflammatory cytokines in bacteria-infected human keratinocytes [24]. These results showed that the anti-inflammatory effect of BV is caused at relatively low concentrations. In the present study, it was shown that the inhibitory effects of BV for inflammatory cytokines were also achieved in a dose-dependent manner, especially at a low concentration of BV.

Administration of melittin, which is a major component of BV, significantly decreased the expression of various inflammatory cytokines in *P. acnes*-treated keratinocytes. In particular, melittin suppressed the expression of TNF-α and IL-1β through regulation of the NF-κB and MAPK signaling pathways in keratinocytes [23]. In the present study, BV suppressed PgLPS-inducible IKK phosphorylation and nuclear NF-κB p65 activation in keratinocytes. Also, the PgLPS-induced ERK MAPK signal and AP-1 activation in keratinocytes was specifically inhibited by BV. Moreover, the binding activity of NF-κB and AP-1 was increased in PgLPS-induced keratinocytes. In contrast, BV markedly withdrew the responses induced by PgLPS in keratinocytes by suppression of inflammatory cytokines through modulation of NF-κB and AP-1 transcription factors. These results support the previous paper that BV might be used for the prevention of progression of inflammatory diseases induced by bacteria [23].

PgLPS is a key factor in the development of periodontitis. Gingival epithelial cells, including keratinocytes, which are the major constituents of gingival connective tissue, may directly interact with *P. gingivalis* and its bacterial products, including LPS, in periodontitis lesions [57,58]. For its part, PgLPS promotes cell proliferation and production of IL-1β, IL-6, and IL-8 [59]. This is in agreement with the findings of Roberts et al. [60], who found that the mononuclear cells derived from the periodontal tissue have the capacity to respond to the periodontal pathogens and their virulence factors and induce the expression of pro- and anti-inflammatory cytokines (IL-1α, IL-1β, IL-6, IL-8, IL-12, IL-13, TNF-α, and IFN-γ) in tissues affected by periodontal disease. In the present study, it was identified that PgLPS-treated keratinocytes showed increased expressions of IL-1β, IL-6, IL-8, TNF-α, and IFN-γ.

Among these cytokines, IL-6 is a pro-inflammatory cytokine which highlights the importance in periodontitis. It was reported that IL-6 induces osteoclastogenesis and alveolar bone resorption in periodontitis [36]. Perlman et al. observed that over-expression of p21 could suppress IL-6 production by reducing the activation of AP-1 [61]. It has been shown that viable *P. gingivalis* could activate AP-1 and then induce the upregulation of IL-6 [62]. In the present study, it was identified that transcription factor AP-1 was related to the expressions of inflammatory cytokines in the PgLPS-treated keratinocytes. The expressions of AP-1 and IL-6 were inhibited by BV treatment. In the present study, it was identified that transcription factors AP-1 and NF-κB were related to the expressions of inflammatory cytokines in the PgLPS-treated keratinocytes. The expressions of these transcription factors were inhibited by BV treatment.

During inflammatory reactions in the skin, IFN-γ has an essential role in host defense against various bacteria by activating phagocytes and inflammatory reactions [63]. TNF-α and IL-8 are well described as pro-inflammatory cytokines induced by bacteria that may play a role in the chemo-attraction and maturation of inflammatory cells [64]. Some papers have reported that LPS and bacteria directly stimulate the production of TNF-α, IL-1β, IL-8, and IFN-γ via TLR expression [65,66]. Thus, it is suggested that inhibition of TLR activation may be a novel and effective therapeutic strategy for increased pro-inflammatory mediators [45,67].

TLRs are involved in host defense against a variety of pathogens including bacteria, parasites, and fungi [45,68]. TLR-4 is known to be activated, as well as inhibited, by PgLPS [69]. TLR-2 is mostly activated by pathogen-associated molecular patterns from gram-positive bacteria, while TLR-4 is more associated with entero-bacterial LPS-activation [70]. This represents another reason why TLR-4 was used for this experiment, because PgLPS was used to injure the keratinocytes for the present study.

The major pathway used by most TLRs leads to the activation of the NF-κB, MAPK, and AP-1 signaling pathways. They then modulate inflammatory gene expression, which is crucial for the innate immune response to inflammation [47,71]. NF-κB comprises a family of inducible transcription factors that serve as important regulators of the host immune and inflammatory responses [72,73]. Other important AP-1 signaling pathways have been implicated in multiple cellular events, such as proliferation, survival, differentiation, and inflammation [50,74]. Activation of MAPK and AP-1 leads to increased production of pro-inflammatory mediators, such as TNF-α and IL-1β [75,76]. In the present study, expression of ERK MAPK and AP-1 was increased by PgLPS, and BV decreased them.

In summary, *P. gingivalis* infection makes a crucial contribution to the upregulation of pro-inflammatory cytokines during infection. PgLPS-treated keratinocytes upregulated the expression of inflammatory cytokines. It has been demonstrated that BV had an anti-inflammatory effect through suppression of pro-inflammatory cytokines. Also, administration of BV significantly decreased the activation of transcription factors—which were related to pro-inflammatory cytokines—in LPS-treated keratinocytes. 

These results demonstrated that bee venom has effects on anti-inflammatory activity against PgLPS-treated keratinocytes. BV suppressed the production of pro-inflammatory cytokines, such as IFN-γ, IL-1β, IL-6, IL-8, and TNF-α, through blocking the prime signaling pathway, including Akt, NF-κB, ERK1/2, and AP1, induced by PgLPS in HaCaT cells. Also, it was demonstrated that BV blocked the transcription factors NF-κB and AP-1, resulting in the suppression of pro-inflammatory cytokines. In addition, the results indicate that BV can protect keratinocytes against PgLPS-mediated injury. This might be the first evidence that BV can be effective for the treatment of *P. gingivalis*-induced periodontitis.

Therefore, it is suggested that bee venom is an alternative treatment to anti-inflammatory therapy for periodontitis. However, the anti-bacterial properties of the bee venom components on *P. gingivalis* were not determined. The precise anti-bacterial mechanisms of the bee venom components require further investigation. These results regarding the regulation of important cytokines in *P. gingivalis* LPS-treated keratinocytes could contribute to further understanding of the pathogenesis of periodontitis and the development of novel preventive and therapeutic approaches to controlling this disease.

## 4. Materials and Methods

### 4.1. Bee Venom

Colonies of natural honey bees used in this study were maintained at the National Institute of Agricultural Science and Technology, Suwon, Korea. BV was collected using a BV collecting device (Chunggin, Korea) in a sterile manner under strict laboratory conditions. In brief, the BV collector was placed in the hive, and the bees were given enough electric shock to cause them to sting a glass plate from which dried BV was later scraped off. The collected BV was purified by the method of Han et al. [77]. In brief, all of the bioactive components of BV used in the experiment were confirmed by size exclusion gel chromatography (AKTAexplorer, Pharmacia, Pleasanton, CA, USA) by dissolving in 0.1 M ammonium formate adjusted to pH 4.5. A Sephadex TM75 column (Amersham Biosciences, Piscataway, NJ, USA) with further purification by a Source 15RPC ST column (GE Healthcare, Little Chalfont, UK) with 0.1% trifluoracetic acid in 20% acetonitrile as the eluent was used to confirm the presence of melittin, the major active ingredient of BV.

### 4.2. Cell Culture and Reagents

A human keratinocyte cell line, HaCaT cells, was cultured in Dulbecco’s Modified Eagle Medium (DMEM) supplemented with 10% fetal bovine serum (FBS) and 1% antibiotics at 37 °C in a humidified 5% CO_2_ incubator. HaCaT cells were seeded at 1.0 × 10^6^ cells per 3 mL complete medium in a 100 mm TC-treated cell culture dish. The medium was changed 24 h later to serum-free medium containing the indicated concentrations of BV (10, 100 and 250 ng/mL; Sigma, St. Louis, MO, USA). After 1 h, the cells were co-treated with 100 ng/mL of PgLPS (InvivoGen, San Diego, CA, USA). After 7 h, the cells and culture supernatants were collected.

### 4.3. Cell Viability Assay

The cell viability of HaCaT was determined by CCK-8 assay (Dojindo, Kumamoto, Japan). The cells were seeded in a 96-well plate at 5.0 × 10^3^ cells/well and pre-incubated for 24 h. After pre-incubation, the cells were treated with BV (10, 100, and 250 ng/mL) for 8 or 24 h. Subsequent to bee venom treatment, 10 μL of WST-8 solution [2-(2-methoxy-4-nitrophenyl)-3-(4-nitrophenyl)-5-(2,4-disulfophenyl)-2*H*-tetrazolium, monosodium salt] was added to each well, and the cells were incubated for an additional 4 h at 37 °C. The cell viability values were measured by absorbance at 450 nm using a microplate reader.

### 4.4. Quantitative Real-Time PCR

RNA was extracted from cultured cells with TRIzol Reagent (Thermo Fisher Scientific, Waltham, MA, USA) according to the manufacturer’s recommendations. Reverse transcription reaction was performed by using AccuPower RT Premix and Oligo dT18 primer (Bioneer, Daejeon, Korea) according to the manufacturer’s recommendations. Real-time PCR was performed in a LightCycler nano System (Roche Applied Science, Mannheim, Germany) by using LightCycler DNA Master SYBR GREEN I (Roche Applied Science). PCR mixtures contained 100 ng of cDNA and 0.5 μM each of forward and reverse primers. The samples were denatured at 95 °C for 10 min, followed by 45 cycles of annealing and extension at 95 °C for 20 s, 60 °C for 20 s, and 72 °C for 20 s. Expression values were normalized to glyceraldehyde 3-phosphate dehydrogenase (GAPDH). Quantitative real-time PCR products were further confirmed by melting curve analysis. The primer sequences were as follows: TNF-α, forward, 5′-AGTGGTGCCAGCCGATGGGTTGT-3′, and reverse, 5′-GCTGAGTTGGTCCCCCTTCTCCAG-3′; IL-8, forward, 5′-TCCAATTCGGGAGACCTCTA-3′, and reverse, 5′-TAGGCATCACTGCCTGTCAA-3′; IL-6, forward, 5′-GGTACATCCTCGACGGCATCT-3′, and reverse, 5′-GTGCCTCTTTGCTGCTTTCAC-3′; GAPDH, forward, 5’-GGAGCCAAAAGGGTCATCAT-3′, and reverse, 5′-GTGATGGCATGGACTGTGGT-3′.

### 4.5. ELISA

The concentrations of IL-1β and TNF-α in the HaCaT cell supernatants were determined by enzyme-linked immunosorbent assay (ELISA) kit, according to the manufacturer’s instructions (R&D Systems, Minneapolis, MN, USA). The optical density was measured at 450 nm in an ELISA reader (BMG Labtech, Ortenberg, Baden-Württemberg, Germany).

### 4.6. Western Blotting

Cells were lysed by using cell lytic™ M (Sigma). After incubation for 30 min on ice, the lysed cells were centrifuged at 12,000 *g*, 4 °C for 10 min. The supernatants were collected, and protein concentration was determined by using Bradford assay (Bio-Rad Laboratories, Hercules, CA, USA). Protein extracts were separated on 8% to 12% SDS-polyacrylamide gels and transferred to nitrocellulose membrane (GE healthcare, Little Chalfont, Buckinghamshire, UK) using the standard SDS-PAGE procedure. The membrane was blocked in 5% (*w*/*v*) skim milk in TBS-T (25 mM Tris, pH 7.4; 3 mM KCL; 140 mM NaCl; and 0.1% (*v*/*v*) Tween-20) for 1 h at room temperature. After blocking, the membrane was probed with primary antibody for 4 h at room temperature and was washed with TBS-T for 10 min on the shaker, repeating the process two times, for a total of three washes. Then, the membrane was probed with horseradish peroxidase (HRP)-conjugated secondary antibody for 2 h at room temperature. Following a repeat of the wash step, the membrane was incubated in enhanced chemiluminescence (ECL) detection reagents (Thermo Fisher Scientific, Waltham, MA, USA) for 1 min. Signal intensity was quantified by an image analyzer (LAS-3000; Fuji, Tokyo, Japan). Primary antibodies used in the present study were purchased from Abcam (anti-IFN-γ, ab9657; anti-TNF-α, ab1793; Cambridge, Cambridgeshire, UK), Cell Signaling Technology (anti-Akt, #9272; anti-phospho-Akt, #9271; anti-ERK, #4695; anti-phospho-ERK, #4377; anti-IκB, #9242; anti-IKK, #2682; anti-phospho-IKK, #2681; anti-NF-κB p65, #3034; anti-phospho-NF-κB p65, #3031; anti-phospho-c-Jun, #9261; Danvers, MA, USA), Santa Cruz Biotechnology (GAPDH, sc-32233; IL-8, sc-8427; TLR-4, sc-10741; Dallas, TX, USA) and Invitrogen (Lamin B1, #33-2000; Carlsbad, CA, USA).

### 4.7. EMSA

Lightshift^®^ Chemiluminescent EMSA Kit (Thermo) was used for the electrophoretic mobility shift assay (EMSA), according to the manufacturer’s instructions. The image analyzer (LAS-3000; Fuji, Tokyo, Japan) was used to detect chemiluminescence of biotin-labeled DNA. The NF-κB (5′-AGTTGAGGGGACTTTCCCAGGC-3′) and AP-1 (5′-CGCTTGATGACTCAGCCGGAA-3′) oligonucleotide probe were end-labeled with DIG-ddUTP.

### 4.8. Immunofluorescence Analysis

Cells were seeded at a density of 3.0 × 10^5^ cells/well in a 4-well chamber slide. After 24 h, the media were changed to serum-free media, containing the 100 ng/mL of BV. After 1 h, the cells were treated with 100 ng/mL of PgLPS (InvivoGen) for 7 h. The treated-cells were washed with PBS and fixed with 4% paraformaldehyde for 20 min at room temperature. Fixed cells were treated with 0.1% Triton X-100 in phosphate-buffered saline (PBS) for 2 min to permeabilize. Following permeabilization, the cells were blocked in PBS, containing 5% bovine serum albumin, at room temperature for 1 h. After blocking, the cells were incubated with diluted primary antibody overnight at 4 °C, and secondary antibody was performed at room temperature for 4 h. The nuclei were stained with Hoechst 33342 solution for 20 min. Slides were mounted by using fluorescence mounting medium (Dako, Santa Clara, CA, USA). Specimens were examined and photographed by the using confocal microscope system (Nikon, Tokyo, Japan). Antibodies used in the present study were the following: anti-TNF-α; anti-phospho-NF-κB (Santa Cruz); anti-mouse-, and anti-rabbit-biotinylated secondary antibodies conjugated with Alexa Fluor 488 or Alexa Fluor 555 (Thermo).

### 4.9. Statistical Analysis

All data are presented as the means ± standard error of the mean (SEM). A Student’s *t*-test was used to assess the significance of independent experiments. Differences with *p* < 0.05 were considered significant.

## References

[1] Liu J., Tang X., Li C., Pan C., Li Q., Geng F., Pan Y. (2015). *Porphyromonas gingivalis* promotes the cell cycle and inflammatory cytokine production in periodontal ligament fibroblasts. Arch. Oral Biol..

[2] Darveau R.P. (2010). Periodontitis: A polymicrobial disruption of host homeostasis. Nat. Rev. Microbiol..

[3] Hajishengallis G. (2015). Periodontitis: From microbial immune subversion to systemic inflammation. Nat. Rev. Immunol..

[4] Lamont R.J., Jenkinson H.F. (2000). Subgingival colonization by *Porphyromonas gingivalis*. Oral Microbiol. Immunol..

[5] Kuboniwa M., Lamont R.J. (2010). Subgingival biofilm formation. Periodontology 2000.

[6] Al Batran R., Al-Bayaty F.H., Al-Obaidi M.M. (2013). In Vivo effect of andrographolide on alveolar bone resorption induced by *Porphyromonas gingivalis* and its relation with antioxidant enzymes. BioMed Res. Int..

[7] Bostanci N., Belibasakis G.N. (2012). *Porphyromonas gingivalis*: An invasive and evasive opportunistic oral pathogen. FEMS Microbiol. Lett..

[8] Andrian E., Grenier D., Rouabhia M. (2006). *Porphyromonas gingivalis*-epithelial cell interactions in periodontitis. J. Dent. Res..

[9] Amano A. (2007). Disruption of epithelial barrier and impairment of cellular function by *Porphyromonas gingivalis*. Front. Biosci. J. Virtual Libr..

[10] Colombo A.V., da Silva C.M., Haffajee A., Colombo A.P. (2007). Identification of intracellular oral species within human crevicular epithelial cells from subjects with chronic periodontitis by fluorescence in situ hybridization. J. Periodontal Res..

[11] Lamont R.J., Yilmaz O. (2002). In or out: The invasiveness of oral bacteria. Periodontology 2000.

[12] Kinane D.F., Galicia J.C., Gorr S.U., Stathopoulou P.G., Benakanakere M.P. (2008). Gingivalis interactions with epithelial cells. Front. Biosci. J. Virtual Libr..

[13] Macpherson A., Zoheir N., Awang R.A., Culshaw S., Ramage G., Lappin D.F., Nile C.J. (2014). The alpha 7 nicotinic receptor agonist pha-543613 hydrochloride inhibits *Porphyromonas gingivalis*-induced expression of interleukin-8 by oral keratinocytes. Inflamm. Res..

[14] Raingeaud J., Pierre J. (2005). Interleukin-4 downregulates tnfalpha-induced il-8 production in keratinocytes. FEBS Lett..

[15] Feliciani C., Gupta A.K., Sauder D.N. (1996). Keratinocytes and cytokine/growth factors. Crit. Rev. Oral Biol. Med..

[16] Tribble G.D., Kerr J.E., Wang B.Y. (2013). Genetic diversity in the oral pathogen *Porphyromonas gingivalis*: Molecular mechanisms and biological consequences. Future Microbiol..

[17] Armitage G.C. (2002). Classifying periodontal diseases—A long-standing dilemma. Periodontology 2000.

[18] Colombo A.P., Bennet S., Cotton S.L., Goodson J.M., Kent R., Haffajee A.D., Socransky S.S., Hasturk H., Van Dyke T.E., Dewhirst F.E. (2012). Impact of periodontal therapy on the subgingival microbiota of severe periodontitis: Comparison between good responders and individuals with refractory periodontitis using the human oral microbe identification microarray. J. Periodontol..

[19] Kornman K.S., Holt S.C., Robertson P.B. (1981). The microbiology of ligature-induced periodontitis in the cynomolgus monkey. J. Periodontal Res..

[20] Brecx M.C., Nalbandian J., Ooya K., Kornman K.S., Robertson P.B. (1985). Morphological studies on periodontal disease in the cynomolgus monkey. Ii. Light microscopic observations on ligature-induced periodontitis. J. Periodontal Res..

[21] Holt S.C., Ebersole J., Felton J., Brunsvold M., Kornman K.S. (1988). Implantation of bacteroides gingivalis in nonhuman primates initiates progression of periodontitis. Science.

[22] Assuma R., Oates T., Cochran D., Amar S., Graves D.T. (1998). Il-1 and tnf antagonists inhibit the inflammatory response and bone loss in experimental periodontitis. J. Immunol..

[23] Lee W.R., Kim K.H., An H.J., Kim J.Y., Chang Y.C., Chung H., Park Y.Y., Lee M.L., Park K.K. (2014). The protective effects of melittin on propionibacterium acnes-induced inflammatory responses in vitro and in vivo. J. Investig. Dermatol..

[24] Kim J.Y., Lee W.R., Kim K.H., An H.J., Chang Y.C., Han S.M., Park Y.Y., Pak S.C., Park K.K. (2015). Effects of bee venom against propionibacterium acnes-induced inflammation in human keratinocytes and monocytes. Int. J. Mol. Med..

[25] Kwon Y.B., Lee J.D., Lee H.J., Han H.J., Mar W.C., Kang S.K., Beitz A.J., Lee J.H. (2001). Bee venom injection into an acupuncture point reduces arthritis associated edema and nociceptive responses. Pain.

[26] Park H.J., Lee S.H., Son D.J., Oh K.W., Kim K.H., Song H.S., Kim G.J., Oh G.T., Yoon D.Y., Hong J.T. (2004). Antiarthritic effect of bee venom: Inhibition of inflammation mediator generation by suppression of nf-kappab through interaction with the p50 subunit. Arthritis Rheum..

[27] Kwon Y.B., Lee H.J., Han H.J., Mar W.C., Kang S.K., Yoon O.B., Beitz A.J., Lee J.H. (2002). The water-soluble fraction of bee venom produces antinociceptive and anti-inflammatory effects on rheumatoid arthritis in rats. Life Sci..

[28] Taylor J.J. (2010). Cytokine regulation of immune responses to *Porphyromonas gingivalis*. Periodontology 2000.

[29] Pathirana R.D., O’Brien-Simpson N.M., Reynolds E.C. (2010). Host immune responses to *Porphyromonas gingivalis* antigens. Periodontology 2000.

[30] Ara T., Kurata K., Hirai K., Uchihashi T., Uematsu T., Imamura Y., Furusawa K., Kurihara S., Wang P.L. (2009). Human gingival fibroblasts are critical in sustaining inflammation in periodontal disease. J. Periodontal Res..

[31] Gursoy U.K., Kononen E., Uitto V.J. (2008). Stimulation of epithelial cell matrix metalloproteinase (mmp-2, -9, -13) and interleukin-8 secretion by fusobacteria. Oral Microbiol. Immunol..

[32] Liu Y.C., Lerner U.H., Teng Y.T. (2010). Cytokine responses against periodontal infection: Protective and destructive roles. Periodontology 2000.

[33] Mans J.J., Hendrickson E.L., Hackett M., Lamont R.J. (2010). Cellular and bacterial profiles associated with oral epithelium-microbiota interactions. Periodontology 2000.

[34] Roberts F.A., Hockett R.D., Bucy R.P., Michalek S.M. (1997). Quantitative assessment of inflammatory cytokine gene expression in chronic adult periodontitis. Oral Microbiol. Immunol..

[35] Seymour G.J., Gemmell E., Kjeldsen M., Yamazaki K., Nakajima T., Hara K. (1996). Cellular immunity and hypersensitivity as components of periodontal destruction. Oral Dis..

[36] Morandini A.C., Chaves Souza P.P., Ramos-Junior E.S., Brozoski D.T., Sipert C.R., Souza Costa C.A., Santos C.F. (2013). Toll-like receptor 2 knockdown modulates interleukin (il)-6 and il-8 but not stromal derived factor-1 (sdf-1/cxcl12) in human periodontal ligament and gingival fibroblasts. J. Periodontol..

[37] Lin S.J., Chen Y.L., Kuo M.Y., Li C.L., Lu H.K. (2005). Measurement of gp130 cytokines oncostatin m and il-6 in gingival crevicular fluid of patients with chronic periodontitis. Cytokine.

[38] Garlet G.P., Avila-Campos M.J., Milanezi C.M., Ferreira B.R., Silva J.S. (2005). Actinobacillus actinomycetemcomitans-induced periodontal disease in mice: Patterns of cytokine, chemokine, and chemokine receptor expression and leukocyte migration. Microbes Infect..

[39] Jain A., Basal E. (2003). Inhibition of propionibacterium acnes-induced mediators of inflammation by indian herbs. Phytomed. Int. J. Phytother. Phytopharmacol..

[40] Kang S., Cho S., Chung J.H., Hammerberg C., Fisher G.J., Voorhees J.J. (2005). Inflammation and extracellular matrix degradation mediated by activated transcription factors nuclear factor-kappab and activator protein-1 in inflammatory acne lesions in vivo. Am. J. Pathol..

[41] Shibata M., Katsuyama M., Onodera T., Ehama R., Hosoi J., Tagami H. (2009). Glucocorticoids enhance toll-like receptor 2 expression in human keratinocytes stimulated with propionibacterium acnes or proinflammatory cytokines. J. Investig. Dermatol..

[42] Webster G.F. (2002). Acne vulgaris. Br. Med. J..

[43] Heymann W.R. (2006). Toll-like receptors in acne vulgaris. J. Am. Acad. Dermatol..

[44] Jugeau S., Tenaud I., Knol A.C., Jarrousse V., Quereux G., Khammari A., Dreno B. (2005). Induction of toll-like receptors by propionibacterium acnes. Br. J. Dermatol..

[45] Kim J., Ochoa M.T., Krutzik S.R., Takeuchi O., Uematsu S., Legaspi A.J., Brightbill H.D., Holland D., Cunliffe W.J., Akira S. (2002). Activation of toll-like receptor 2 in acne triggers inflammatory cytokine responses. J. Immunol..

[46] Kim J. (2005). Review of the innate immune response in acne vulgaris: Activation of toll-like receptor 2 in acne triggers inflammatory cytokine responses. Dermatology.

[47] Hari A., Flach T.L., Shi Y., Mydlarski P.R. (2010). Toll-like receptors: Role in dermatological disease. Mediat. Inflamm..

[48] Pivarcsi A., Bodai L., Rethi B., Kenderessy-Szabo A., Koreck A., Szell M., Beer Z., Bata-Csorgoo Z., Magocsi M., Rajnavolgyi E. (2003). Expression and function of toll-like receptors 2 and 4 in human keratinocytes. Int. Immunol..

[49] Kollisch G., Kalali B.N., Voelcker V., Wallich R., Behrendt H., Ring J., Bauer S., Jakob T., Mempel M., Ollert M. (2005). Various members of the toll-like receptor family contribute to the innate immune response of human epidermal keratinocytes. Immunology.

[50] Grange P.A., Raingeaud J., Calvez V., Dupin N. (2009). Nicotinamide inhibits propionibacterium acnes-induced il-8 production in keratinocytes through the nf-kappab and mapk pathways. J. Dermatol. Sci..

[51] Park S.Y., Park D.J., Kim Y.H., Kim Y., Choi Y.W., Lee S.J. (2011). Schisandra chinensis alpha-iso-cubebenol induces heme oxygenase-1 expression through pi3k/akt and nrf2 signaling and has anti-inflammatory activity in *Porphyromonas gingivalis* lipopolysaccharide-stimulated macrophages. Int. Immunopharmacol..

[52] Bodet C., La V.D., Gafner S., Bergeron C., Grenier D. (2008). A licorice extract reduces lipopolysaccharide-induced proinflammatory cytokine secretion by macrophages and whole blood. J. Periodontol..

[53] Rahman M.M., McFadden G. (2011). Modulation of nf-kappab signalling by microbial pathogens. Nat. Rev. Microbiol..

[54] Bozinovski S., Jones J.E., Vlahos R., Hamilton J.A., Anderson G.P. (2002). Granulocyte/macrophage-colony-stimulating factor (gm-csf) regulates lung innate immunity to lipopolysaccharide through akt/erk activation of nfkappa b and ap-1 in vivo. J. Biol. Chem..

[55] Palm N.W., Medzhitov R. (2013). Role of the inflammasome in defense against venoms. Proc. Natl. Acad. Sci. USA.

[56] Dombrowski Y., Peric M., Koglin S., Kaymakanov N., Schmezer V., Reinholz M., Ruzicka T., Schauber J. (2012). Honey bee (apis mellifera) venom induces aim2 inflammasome activation in human keratinocytes. Allergy.

[57] Tada H., Matsuyama T., Nishioka T., Hagiwara M., Kiyoura Y., Shimauchi H., Matsushita K. (2016). *Porphyromonas gingivalis* gingipain-dependently enhances il-33 production in human gingival epithelial cells. PLoS ONE.

[58] de Camargo Pereira G., Guimaraes G.N., Planello A.C., Santamaria M.P., de Souza A.P., Line S.R., Marques M.R. (2013). *Porphyromonas gingivalis* lps stimulation downregulates dnmt1, dnmt3a, and jmjd3 gene expression levels in human hacat keratinocytes. Clin. Oral Investig..

[59] Kato H., Taguchi Y., Tominaga K., Umeda M., Tanaka A. (2014). *Porphyromonas gingivalis* lps inhibits osteoblastic differentiation and promotes pro-inflammatory cytokine production in human periodontal ligament stem cells. Arch. Oral Biol..

[60] Roberts F.A., McCaffery K.A., Michalek S.M. (1997). Profile of cytokine mrna expression in chronic adult periodontitis. J. Dent. Res..

[61] Perlman H., Bradley K., Liu H., Cole S., Shamiyeh E., Smith R.C., Walsh K., Fiore S., Koch A.E., Firestein G.S. (2003). Il-6 and matrix metalloproteinase-1 are regulated by the cyclin-dependent kinase inhibitor p21 in synovial fibroblasts. J. Immunol..

[62] Yanti, Lee M., Kim D., Hwang J.K. (2009). Inhibitory effect of panduratin a on c-jun n-terminal kinase and activator protein-1 signaling involved in *Porphyromonas gingivalis* supernatant-stimulated matrix metalloproteinase-9 expression in human oral epidermoid cells. Biol. Pharm. Bull..

[63] Kawa K., Tsutsui H., Uchiyama R., Kato J., Matsui K., Iwakura Y., Matsumoto T., Nakanishi K. (2010). Ifn-gamma is a master regulator of endotoxin shock syndrome in mice primed with heat-killed propionibacterium acnes. Int. Immunol..

[64] Schmidt N., Gans E.H. (2011). Tretinoin: A review of its anti-inflammatory properties in the treatment of acne. J. Clin. Aesthet. Dermatol..

[65] Vowels B.R., Yang S., Leyden J.J. (1995). Induction of proinflammatory cytokines by a soluble factor of propionibacterium acnes: Implications for chronic inflammatory acne. Infect. Immun..

[66] Basal E., Jain A., Kaushal G.P. (2004). Antibody response to crude cell lysate of propionibacterium acnes and induction of pro-inflammatory cytokines in patients with acne and normal healthy subjects. J. Microbiol..

[67] Ryan K.A., Smith M.F., Sanders M.K., Ernst P.B. (2004). Reactive oxygen and nitrogen species differentially regulate toll-like receptor 4-mediated activation of nf-kappa b and interleukin-8 expression. Infect. Immun..

[68] Netea M.G., van der Graaf C., Van der Meer J.W., Kullberg B.J. (2004). Toll-like receptors and the host defense against microbial pathogens: Bringing specificity to the innate-immune system. J. Leukoc. Biol..

[69] Teng Y.T. (2006). Protective and destructive immunity in the periodontium: Part 1—Innate and humoral immunity and the periodontium. J. Dent. Res..

[70] Aderem A., Ulevitch R.J. (2000). Toll-like receptors in the induction of the innate immune response. Nature.

[71] Miggin S.M., O’Neill L.A. (2006). New insights into the regulation of tlr signaling. J. Leukoc. Biol..

[72] Yaron A., Hatzubai A., Davis M., Lavon I., Amit S., Manning A.M., Andersen J.S., Mann M., Mercurio F., Ben-Neriah Y. (1998). Identification of the receptor component of the ikappabalpha-ubiquitin ligase. Nature.

[73] Spencer E., Jiang J., Chen Z.J. (1999). Signal-induced ubiquitination of ikappabalpha by the f-box protein slimb/beta-trcp. Genes Dev..

[74] Duesbery N.S., Webb C.P., Leppla S.H., Gordon V.M., Klimpel K.R., Copeland T.D., Ahn N.G., Oskarsson M.K., Fukasawa K., Paull K.D. (1998). Proteolytic inactivation of map-kinase-kinase by anthrax lethal factor. Science.

[75] Kim J.H., Kim D.H., Baek S.H., Lee H.J., Kim M.R., Kwon H.J., Lee C.H. (2006). Rengyolone inhibits inducible nitric oxide synthase expression and nitric oxide production by down-regulation of nf-kappab and p38 map kinase activity in lps-stimulated raw 264.7 cells. Biochem. Pharmacol..

[76] Moon D.O., Park S.Y., Lee K.J., Heo M.S., Kim K.C., Kim M.O., Lee J.D., Choi Y.H., Kim G.Y. (2007). Bee venom and melittin reduce proinflammatory mediators in lipopolysaccharide-stimulated bv2 microglia. Int. Immunopharmacol..

[77] Han S.M., Kim J.M., Hong I.P., Woo S.O., Kim S.G., Jang H.R., Pak S.C. (2016). Antibacterial activity and antibiotic-enhancing effects of honeybee venom against methicillin-resistant staphylococcus aureus. Molecules.

